# Supplementary material for: Inhomogeneous high temperature melting and decoupling of charge density waves in spin-triplet superconductor UTe2
Source: Nat Commun. 2024 May 25;15:4456. doi: 10.1038/s41467-024-48844-7 (PMC11127989; doi:10.1038/s41467-024-48844-7)
Supplement: Supplementary file 1 — Supplementary Information [file 41467_2024_48844_MOESM1_ESM.pdf]

Supplementary Information for "Inhomogeneous high temperature melting and decoupling of charge density waves in spin-triplet superconductor UTe_2"

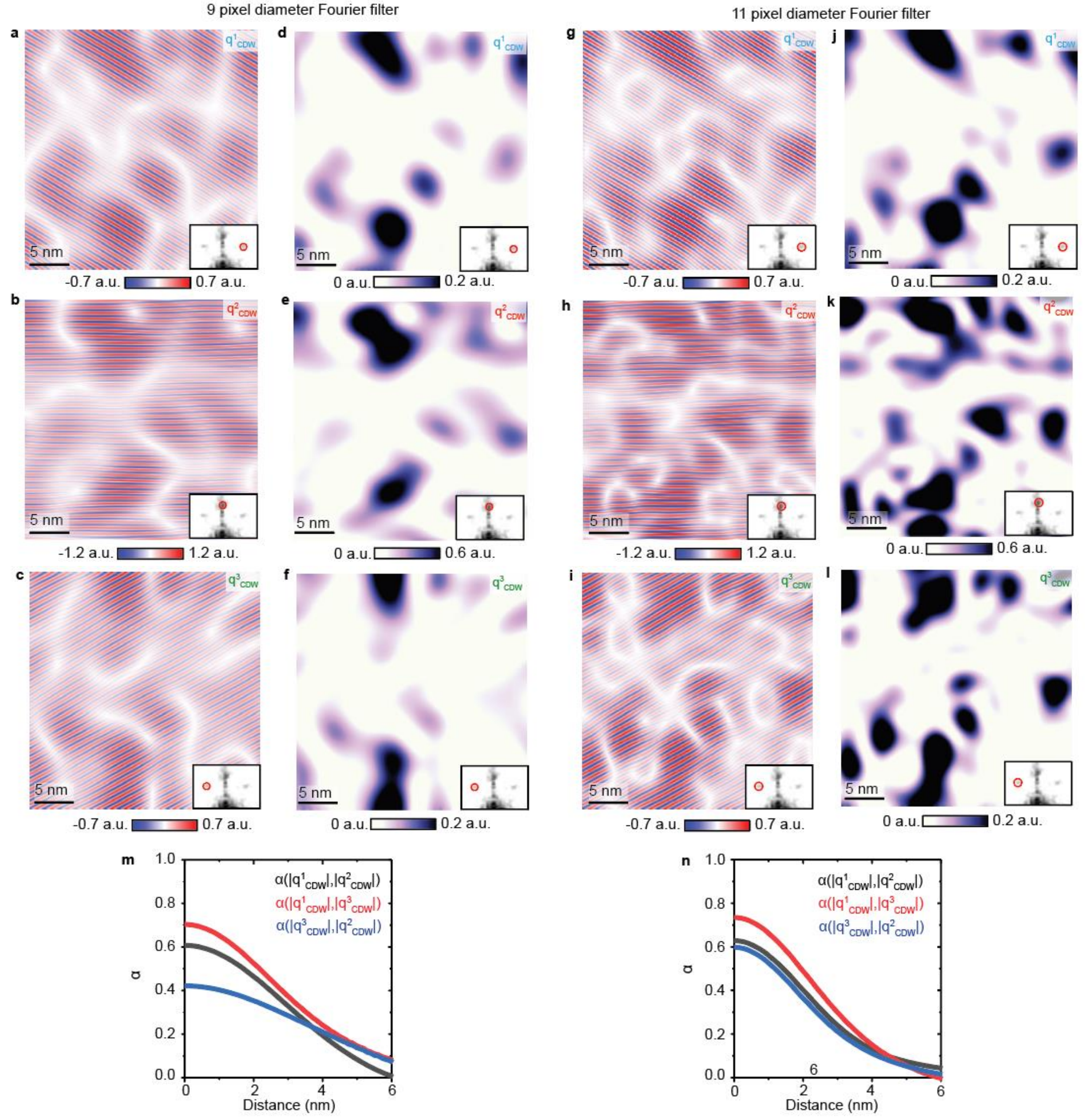

Supplementary Figure 1. Investigating the sensitivity of the CDW amplitude maps on the size of the filtering window. (a-c) Fourier filtered integrated dI/dV maps from Figure 3 using a 9 pixel diameter (in Fourier space) around (a) q^1_{CDW}, (b) q^2_{CDW} and (c) q^3_{CDW}. (d-f) Amplitude maps extracted from (a-c). (g-l) Images equivalent to those in (a-f) but this time using an 11 pixel diameter (in Fourier space) around each CDW peak. Insets in (a-l) represent relevant portions of the Fourier transform of integrated dI/dV map. The size of the filtering window used is denoted by red circles in each inset. (m,n) Cross-correlation coefficients between the three CDW amplitude maps: (m) for maps in (d-f), and (n) for maps in (j-l).

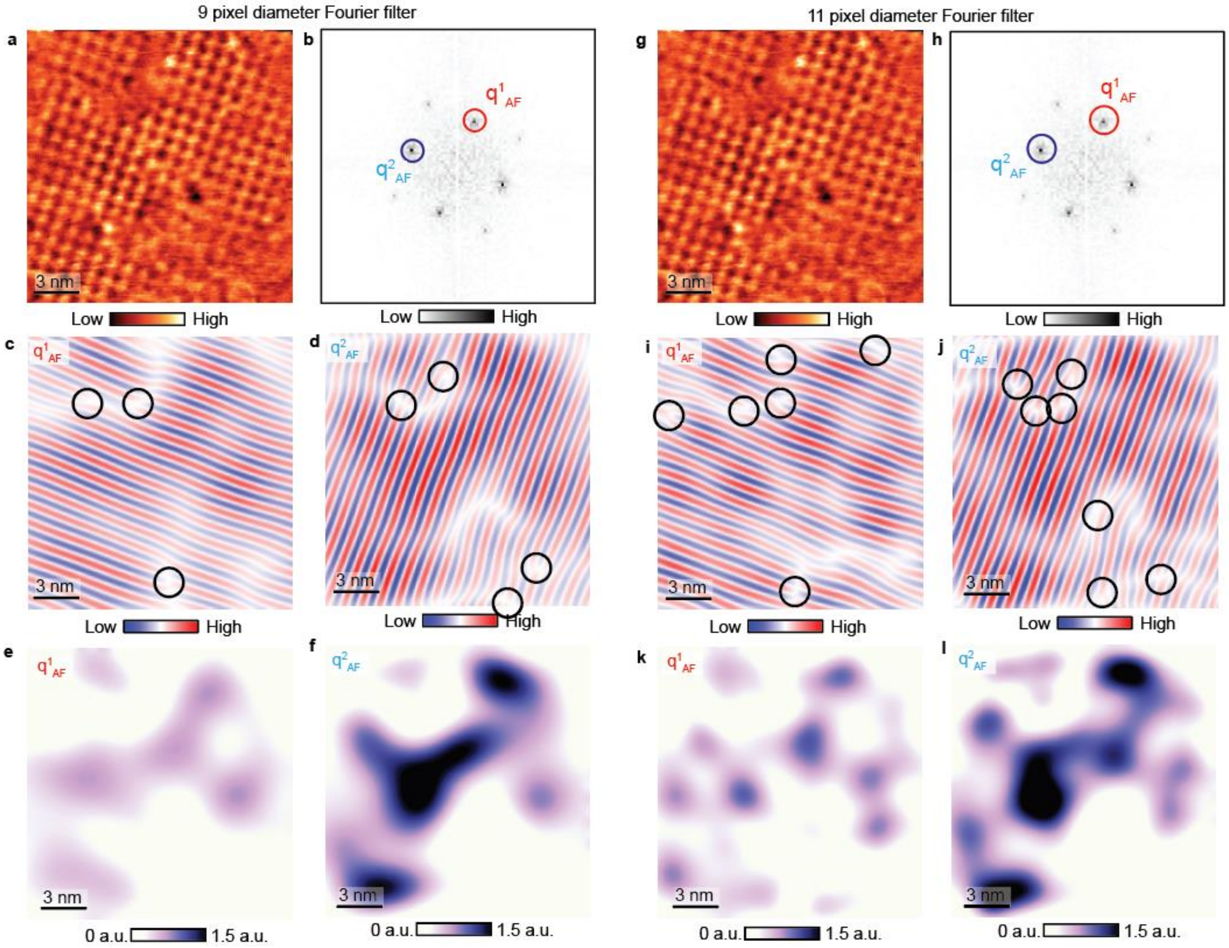

Supplementary Figure 2. Investigating Fourier filtering of inhomogeneous antiferromagnetic order in La-doped Sr_2IrO_4. (a) Spin-polarized STM image showing checkerboard-like pattern originating from local antiferromagnetic (AF) ordering in La-doped Sr_2IrO_4 [1]. (b) Fourier transform (FT) of image in (a) showing two AF peaks circled as q^1_{AF} and q^2_{AF}. AF order is spatially inhomogeneous as it can be seen in (a), which results in diffuse AF peaks in the FT in (b). (c,d) Fourier filtered image in (a) using a 9 pixel diameter (in Fourier space) around (c) q^1_{AF} or (d) q^2_{AF}. (e-f) Amplitude maps extracted from (c-d). (g-l) Images equivalent to those in (a-f) but this time using an 11 pixel diameter (in Fourier space) around each AF peak. The size of the filtering window used is denoted by red circles in (b,h). As it can be seen, AF amplitude maps are all very similar (e,f,k,l), while the dislocation-like feature in filtered data (c,d,i,j) appear to shift around and/or disappear/nucleate. Data from Zhao et al, *Nature Physics* 15, 1267 (2019).

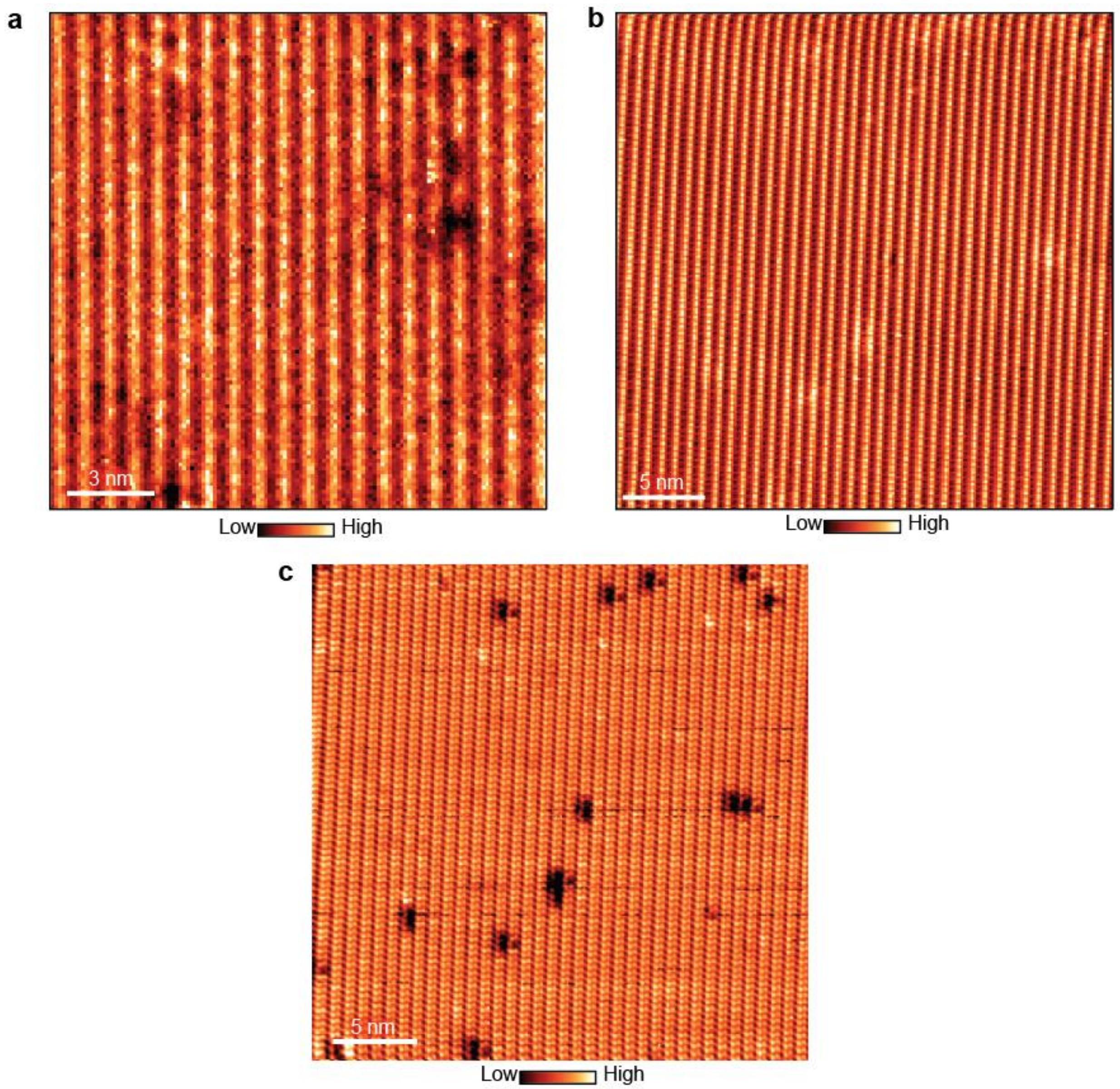

Supplementary Figure 3. Raw dI/dV maps from main text figures. Raw dI/dV maps from (a) Figure 1, (b) Figure 5, and (c) Figure 3. STM setup condition: V_{sample} = 20 mV, I_{set} = 40 pA, V_{exc} = 3 mV (a); V_{sample} = -50 mV, I_{set} = 300 pA, V_{exc} = 10 mV (b); V_{sample} = 50 mV, I_{set} = 300 pA, V_{exc} = 10 mV (c).

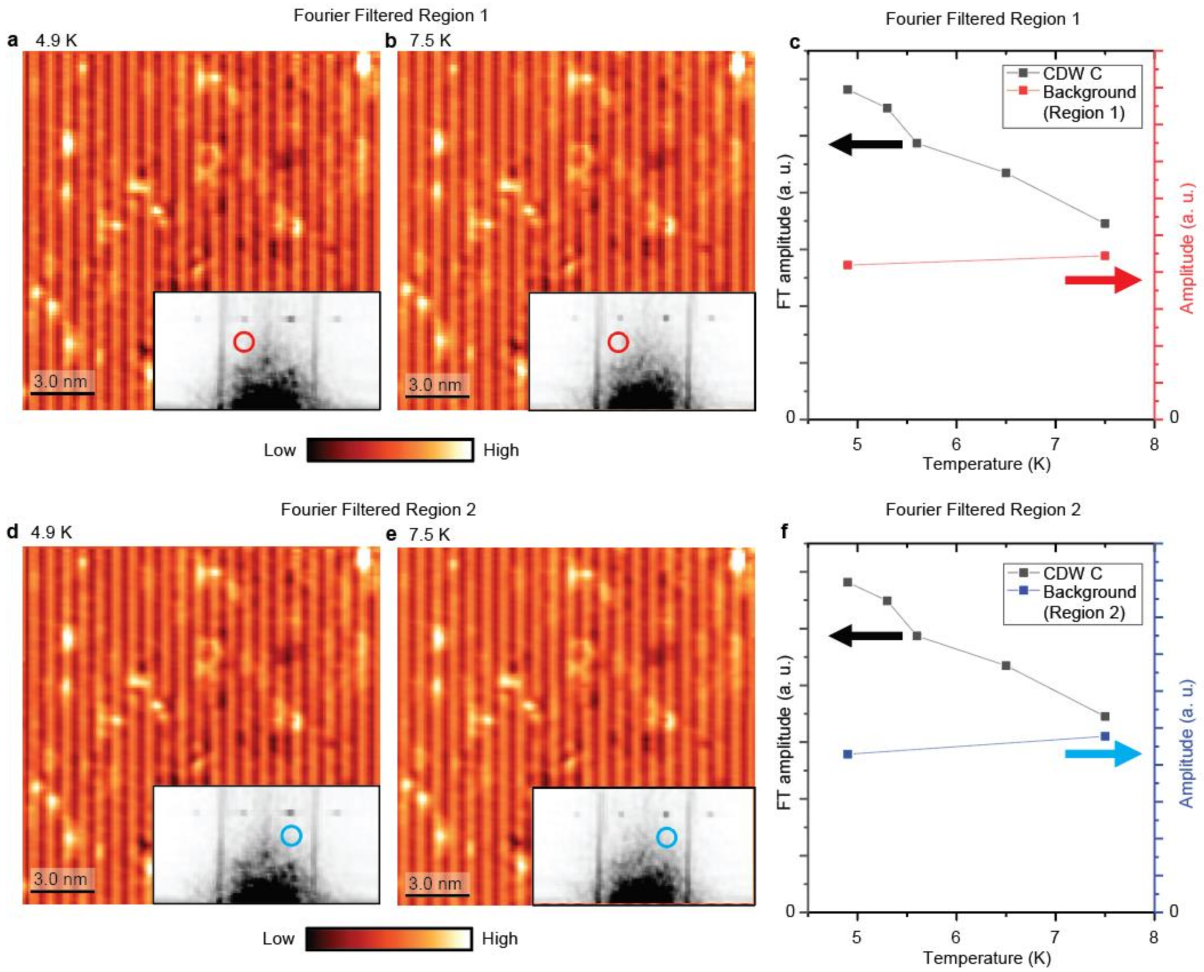

Supplementary Figure 4. Temperature dependence of the Fourier amplitudes of the CDW peaks vs randomly selected background signals. (a-b) dI/dV map at 4.9 K and 7.5 K, respectively, and the corresponding Fourier transform inset from Figures 2 and 4. (c) Average intensity of the Fourier filter amplitude map generated by region 1 (circled in red in the Fourier transform inset). For comparison, in black, we show a representative amplitude trend of a CDW peak being suppressed with temperature. (d-e) dI/dV maps at 4.9 K and 7.5 K, respectively, and corresponding Fourier transform inset for region 2, in blue. (f) Average intensity of the Fourier filter amplitude map generated by the background region 2, which is again temperature-independent.

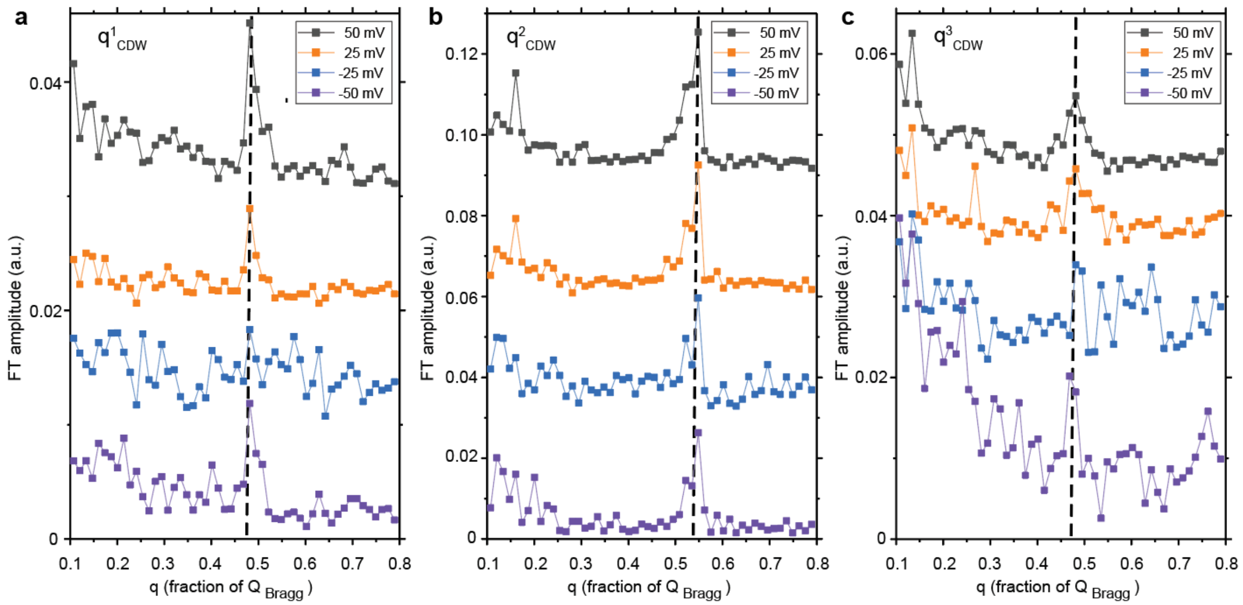

Supplementary Figure 5. Absence of energy dispersion of the CDW peaks. (a-c) Energy dependent linecuts of the Fourier transform of dI/dV maps from Figure 3 along the (a) q^1_{CDW}, (b) q^2_{CDW} and (c) q^3_{CDW} directions. The lack of dispersion in the peaks from -50 mV to +50 mV presents another piece of evidence that these peaks are not due to quasiparticle interference.

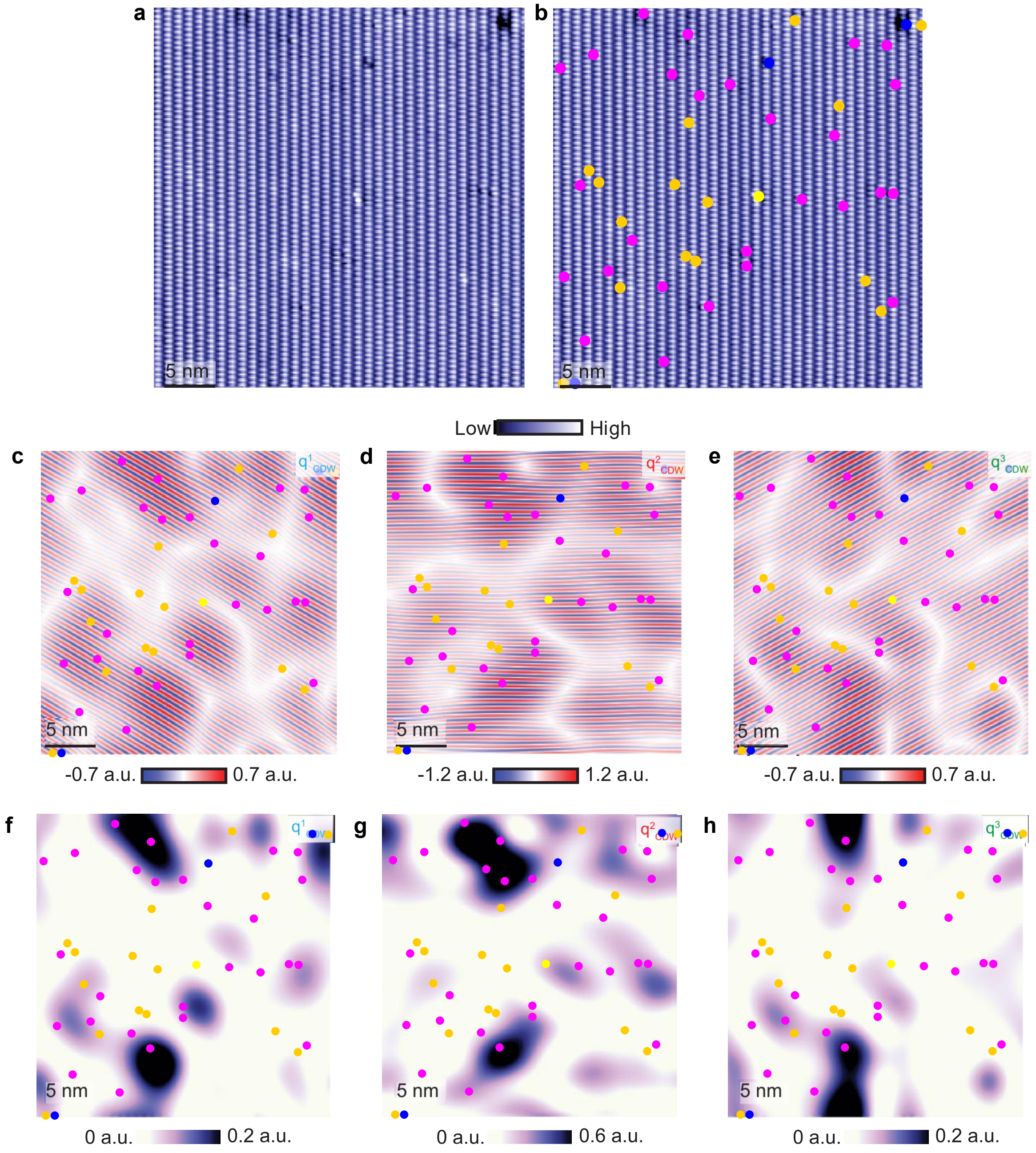

Supplementary Figure 6. Investigating CDW inhomogeneity around local disorder. (a) STM topograph and (b) the same topograph with four types of impurities superimposed in different colors. Impurities are categorized based on their spatial signature in the topograph. (c-e) Fourier filtered integrated dI/dV maps from Figure 3 using a 9-pixel diameter (in Fourier space) around (c) q^1_{CDW}, (d) q^2_{CDW} and (e) q^3_{CDW} with impurity locations superimposed. (f-h) Amplitude maps extracted from (c-e) with impurity locations superimposed. Visual inspection leads us to conclude that there is no substantial correlation between impurity locations and CDW inhomogeneity.
